# Developing fit-for-purpose funding models for rural settings: Lessons from the evaluation of a step-up/step-down service in regional Australia

**DOI:** 10.3389/fpsyt.2023.1036017

**Published:** 2023-01-26

**Authors:** Mathew Coleman, Beatriz Cuesta-Briand, Hanh Ngo, Rachel Bass, Naomi Mills-Edward, Priscilla Ennals

**Affiliations:** ^1^The Rural Clinical School of WA, The University of Western Australia, Albany, WA, Australia; ^2^Great Southern Mental Health Service, WA Country Health Service, Albany, WA, Australia; ^3^Telethon Kids Institute, Nedlands, WA, Australia; ^4^Neami National, Albany, WA, Australia; ^5^Neami National, Burswood, WA, Australia; ^6^Neami National, Preston, VIC, Australia

**Keywords:** funding model, rural settings, rural disparities, service evaluation, step-up/step-down care, community mental health

## Abstract

**Introduction:**

Sub-acute mental health community services provide a bridging service between hospital and community care. There is limited understanding of the local factors that influence success, and of the funding implications of delivering services in rural areas.

**Methods:**

This paper draws from quantitative and qualitative evaluation data from a regional Western Australian service to explore these issues.

**Results:**

Consumers satisfaction with the service was high and, overall, admission to the service resulted in positive outcomes. High re-admission rates may be linked to limited community support services following discharge.

**Discussion:**

Our results suggest that outcomes may be enhanced by implementing flexible approaches that address the resource limitations of the rural context, and that the current funding model for sub-acute mental health services in rural Australian may not be fit for purpose. More needs to be understood about how these services can be better integrated with existing support services, and how they can be better funded.

## 1. Introduction

In the wake of deinstitutionalization, many countries have worked toward developing responsive, community-based mental health service delivery models (1). In Australia, a national review of mental health programs and services highlighted the need to strengthen the sector’s ability to provide services which keep people out of hospital and emergency departments, and enable them to lead contributing lives in the community (2). In its response to the review, the Australian Government recognized that directing more attention toward prevention, early intervention and a continuous pathway to recovery would reduce the need for high intensity and costly interventions (3).

Sub-acute mental health community services provide a “bridging service,” operating as early intervention from community-based care (step-up) or transition from hospital to the community (step-down) (4), and have gradually become embedded in the Australian mental health service system (5). Step Up/Step Down (SUSD) services follow a recovery-oriented model (6), and are characterized by a shift from clinical recovery to “personal recovery” (7), described as a uniquely individual process involving gaining new meaning and purpose in life and sometimes a reformation of identity, even in the presence of on-going symptoms (8).

Given the diversity of experiences and outcomes for people moving from illness to wellness, the adoption of a personal recovery approach provides challenges for evidence-based practice, as it is not easily measured or scientifically scrutinized (9). Despite its uptake in several countries, understanding of the SUSD model, or of stepped care in general, in terms of utility, scope, effectiveness and cost, remains limited (4, 10). In Australia, developing an evidence base is further complicated by the heterogeneity within the SUSD sector, with variations relating to governance, commissioning and funding arrangements (operational collaboration or sub-contract agreement between the local health authority and the SUSD service), physical environment (adapted or purpose built), staffing (mix of clinical and psychosocial staff), service delivery model (typically a combination of group and individual programs), length of stay, intended outcomes and performance monitoring (4). In Western Australia (WA), mental health services are purchased by the Mental Health Commission (MHC) from a range of providers including public health service providers, non-government organizations (NGOs) and private service providers (11). The MHC’s commissioning role involves the planning, purchasing, managing, monitoring and evaluating of mental health services in WA, and is implemented through a Procurement Schedule that maps out the procurement timelines for its program areas, with the MHC advertising invitations to tender for its services and interested organizations submitting their proposals (12).

Despite its complexity, there is a growing body of literature that demonstrates the clinical and cost-effectiveness of the SUSD model (1). The evaluation of the growing SUSD sector in Australia has shown positive outcomes regarding quality of life, symptoms and functioning (4, 7, 13–15), progress toward achieving goals identified on the individual recovery plans (16), and consumer satisfaction with service delivery, relating to elements such as service approach and ethos, program activities, safety, and communal qualities and environment (7, 14, 17, 18). Although more limited, there is also some encouraging evidence of the positive short-term impact on quality of life after discharge (15).

Although there is growing evidence on the effectiveness of SUSD services, there is limited understanding of the contextual factors at the local service systems level that can limit or enhance success. This is particularly salient for rural communities, where health care delivery is presented with a set of challenges, including: increased costs of care, challenges in the recruitment and retention of appropriately qualified staff, higher prevalence of chronic health conditions, and attitudes and values that may hinder health-seeking behavior (19). Furthermore, the implementation of SUSD services in rural settings needs to take into account the impact of “rural adversity,” that is, the cycle of adverse events impacting the rural environment (20, 21), recognizing the structural and cultural factors that drive poorer rural mental health outcomes (22). There is some evidence on the effectiveness of SUSD services in regional areas (16); however, to our knowledge, the commissioning implications of delivering sub-acute mental health services in rural areas have not been explored.

This paper draws from evaluation data from a regional SUSD service in WA to illustrate some of the challenges surrounding community mental health service delivery in rural settings and reflect on the service commissioning implications.

## 2. Materials and methods

### 2.1. The service and its context

The service opened in October 2018 as a short-term residential service for people aged 16 years and over, and is operated by a national NGO. The service is in a regional city located approximately 400 km southeast of the state capital Perth. The service has a capacity of six beds, and it is the only of its kind in a region with a catchment area of over 39,000 km^2^ and approximately 60,000 people (23).

The service is funded by the MHC, and offers recovery-focused individual and group programs to support consumers toward identifying their personal strengths and working toward their recovery goals. MHC funding allows for up to 28 days of support, although extensions may be approved. In line with the National Framework for Recovery Oriented Practice (24), the service aims to respond to the diverse life circumstances of people who use it by promoting a culture of hope, optimism, and collaboration, whilst supporting recovery, mental health self-management, and pathways to social inclusion. All aspects of service delivery practice are underpinned by the evidence-based Collaborative Recovery Model framework of care (25).

The service is staffed by a team of Community Rehabilitation Support Workers, Peer Support Workers, Senior Practice Leaders and a Service Manager. Clinical support by a nurse, including clinical governance and escalation of clinical issues to the community mental health team, is provided by the State Government’s Western Australia Country Health Service (WACHS). The clinical nurse is available 7 days a week, 8 h a day, and is an integrated member of the team.

### 2.2. Evaluation objectives and design

The evaluation had three objectives: (1) to describe the profile of consumers accessing the service since its opening; (2) to measure consumers’ recovery outcomes and satisfaction with the service; and (3) to describe the lived experiences of former consumers.

The evaluation had a mixed-methods design, with a quantitative component addressing objectives (1) and (2), and a qualitative component addressing objective (3), and providing a deeper understanding of quantitative findings through the exploration of users’ lived experience and their perspective of the impact of the service. Further, the research adopted a consumer participation approach, with a peer researcher with lived experience of the service forming part of the research team and contributing to the design of the qualitative component and the interpretation of qualitative and overall results.

### 2.3. Data collection

Data for the quantitative component were extracted from routinely collected administrative and assessment data. Customer profile data included: age; gender; country of birth; indigenous status; distance from service; diagnosis; and referral pathway (step-up vs. step-down). Assessment data included entry and exit scores from three self-report instruments: (1) the 10-item Kessler Psychological Distress Scale (K10), designed to measure anxiety and depression, with total scores ranging from 10 to 50 and higher scores indicating greater stress (26); (2) the five-item Work and Social Adjustment Scale (WSAS), measuring impairment in functioning, with total scores ranging from 0 to 40 and higher scores indicating higher levels of impairment (27); and (3) the 10-item General Self-Efficacy Scale (GSES), designed to measure the strength of a person’s belief in their ability to complete tasks and achieve goals, with total scores ranging from 10 to 40 and higher scores indicating higher perceived self-efficacy (28). Data on consumer satisfaction were extracted from the Service Satisfaction Exit Questionnaire completed by consumers upon completion of their stay.

Data for the qualitative component were primarily collected from semi-structured interviews with former consumers conducted in October 2020. Qualitative data gathered through the three open-ended questions from the Exit Questionnaires were also included. Interview participants were recruited by a peer worker not associated with the service from a database of consumers who had used the service since its opening. Those interested in participating received a consent form and information sheet and were asked to provide their contact details so that the interviewer (a member of the research team) could contact them to arrange an interview. The interview schedule consisted of a series of open-ended questions exploring the following topics: (1) circumstances leading to admission; (2) admission process; (3) daily life at the service; (4) interactions with staff and peers; (5) discharge process; (6) support beyond discharge; and (7) life since leaving the service. Basic socio-demographic and clinical data were collected separately before the interview. Interviews were conducted either on the phone or face-to-face and at participants’ venue of choice. Consent was obtained prior to the interview. Interviews were audio-recorded in most cases; alternatively, extensive notes were taken (two participants did not consent to the interview being recorded). In recognition of their time and contribution, and consistent with organizational policy on consumer involvement, participants’ contribution was acknowledged with an AUD40 supermarket voucher.

### 2.4. Data analysis

#### 2.4.1. Quantitative component

For the quantitative component, descriptive summary data were calculated for demographic and clinical data (mean and range for continuous data, and frequency and percentage for categorical data). Pre-post changes in consumers’ scores for each of the three scales (K-10, GSES, and WSAS) were assessed using the paired *t*-test. Consumers’ satisfaction with service was summarized in stacked vertical bar charts. Statistical analyses were performed using the Statistical Analysis System (version 9.4) software (29). Statistical significance was set at α = 0.05.

#### 2.4.2. Qualitative component

For the qualitative component, interview transcripts and notes were imported into NVivo (30) and subjected to thematic analysis within each of the topics explored in the interview. BCB and MC read the transcripts and performed the initial coding, and this was refined in iterative consultation with RB. MC, BCB, HN, and RB all agreed on the main themes. Rigor was enhanced through the iterative process of data collection and analysis, the involvement of the peer researcher in the interpretation of results, data triangulation using the qualitative findings from the Exit Questionnaire, and the contextualization of former service users’ experiences.

### 2.5. Ethics

Ethics clearance was granted by the University of Western Australia Human Research Ethics Committee (Ref RA/4/20/6418) and the service Research and Evaluation Committee.

## 3. Results

### 3.1. Consumer profile

A total of 84 consumers used the service during the period October 2018 to October 2020, and there were 135 episodes of care (admissions). A total of 54 consumers (64.3%) had a single admission whilst 30 (35.7%) had repeat admissions and accounted for 81 (60.0%) of the total episodes of care. Of the 135 admissions, step-up (*n* = 68, 50.4%) and step-down (*n* = 63, 46.7%) referral pathways were roughly evenly distributed, and each admission had an average duration of approximately 22 days.

Consumers’ age ranged from 17 to 64 years, with a median of 40 years. As seen in Table 1, the majority of consumers were female (*n* = 58, 69.0%), non-Indigenous (*n* = 75, 89.3%), and born in Australia (*n* = 73, 86.9%). Consumers with depression (*n* = 22, 26.2%) and personality disorders (*n* = 16, 19.0%) together accounted for almost half of the service users.

**TABLE 1:** Characteristics of 84 consumers accessing Neami Albany service (total 135 admission episodes) between October 2018–2020.

	Characteristics		Frequency	*%*
				SD13.8.
Demographic factors	Age (in years)^†^		Mean 40.8	Range 17-64
	Age group	<=40	43	51.2%
		>40	41	48.8%
	Gender	Male	26	31.0%
		Female	58	69.0%
	Indigenous status	Yes	7	8.3%
		No	75	89.3%
		Missing	2	2.4%
	Distance from residence to service	<=50 kms	53	63.1%
		>50 kms	31	36.9%
	Country of birth	Australia	73	86.9%
		Overseas*	9	10.7%
		Missing	2	2.4%
Systemic factor	Referral type^†^	Step Up	68	50.4%
		Step Down	63	46.7%
		Missing	4	3.0%
Clinical factors	Primary diagnosis	Schizophrenia spectrum disorders	13	15.5%
		Bipolar disorder	8	9.5%
		Personality disorder	16	19.0%
		Depression	22	26.2%
		Anxiety	14	16.7%
		Other psychiatric disorder	7	8.3%
		Not known/Missing	4	4.8%
	Unique patients	Single admission	54	64.3%
		Repeat admissions (up to 5)	30	35.7%
	Length of stay (in days)^†^^‡^		Mean 22.5	SD10.0
				Range 1-44

Information is presented for unique consumers and reflects the most recent episode (according to how Neami records and stores data). This is with the exception for length of stay, which is reported for episodes, not consumers.

^†^Indicates continuous characteristics, hence mean, standard deviation (SD), and range are reported (instead of frequency and % being reported for categorical characteristics). The mean is the average value of a data distribution; whereas the standard deviation and the range show the spread/variation in the data.

*Included: New Zealand, United Kingdom, Scotland, and Papua New Guinea.

^‡^Statistics reported for episodes, not unique consumers.

### 3.2. Consumer recovery outcomes

As seen in Table 2, consumers reported significant improvements in distress levels (reduced K10 scores), general self-efficacy (increased GSE scores), and work and social adjustment (reduced WSAS scores). Improvements were the highest for K10 and WSAS scores (27.2 and 25.9% respectively, compared to baseline) and lower for GSE (17.6% compared to baseline).

**TABLE 2:** Summary of scores on: Kessler Psychological Distress Scale (K-10), General Self-Efficacy Scale (GSE), and Social and Work Adjustment Scale (WSAS)–Entire sample (*n* = 84).

		Entry/Baseline		Exit		Score change				
**n pairs**		**Mean**	**SD**	**Mean**	**SD**	**Mean**	**LL**	**UL**	***p*-value**	**% change relative to Baseline**
K-10	55	33.1	8.5	24.0	7.4	9.0	7.1	11.0	<0.0001	27.2%
GSES	50	23.8	5.7	28.0	6.2	4.2	2.7	5.7	<0.0001	17.6%
WSAS	55	26.3	7.9	19.6	8.6	6.8	4.0	9.5	<0.0001	25.9%

Results in bold font denote statistical significance at α = 0.05. LL, lower limit; UL, upper limit.

Analysis of recovery outcomes for repeat consumers also showed significant, albeit somewhat more modest, improvements in the three scores (data not shown).

### 3.3. Satisfaction with service

**FIGURE 1:**
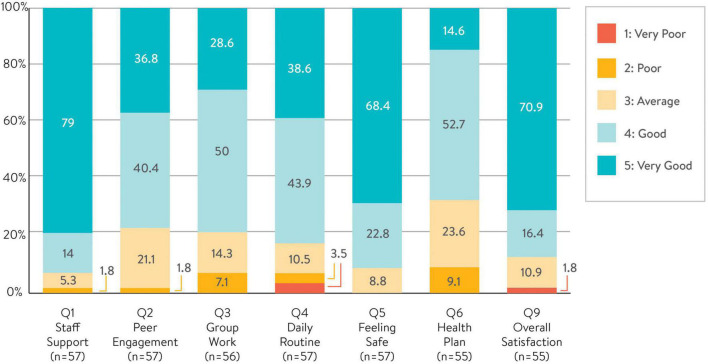
Consumers’ satisfaction with service (sample sizes varies for each question).

Exit Questionnaire data were available for 57 admissions and corresponded to 57 unique consumers. As seen in Figure 1, overall consumers’ satisfaction with the service was high. Across all the seven Likert-scale items (Qs1–6, and Q9), responses to Staff Support (Q1) were overwhelmingly positive with 93% (*n* = 53) rating this item Good or Very Good, followed by Safety (Q5) with 91% (*n* = 52), and Overall Satisfaction (Q9) with 87% (*n* = 50). The level of confidence in using the Health Plan appeared to be the least favored, with 67% (*n* = 38) rating this item positively.

Consumers’ satisfaction with the service in relation to Staff Support, Peer Engagement and Feeling Safe strongly correlated with some, but not all, change outcomes (data not shown). This suggests that the existing questionnaire may have limited ability in accurately reflecting recovery outcomes.

### 3.4. Consumers’ lived experience

We interviewed a total of 14 former service users. As seen in Table 3, our sample had more males compared with administrative (quantitative) consumer profile data, whilst data on age group are roughly consistent with administrative data. Depression was the most commonly reported diagnosed mental health condition. Self-reported data suggest that we interviewed more consumers with multiple admissions compared with administrative data, and also more participants with a step-up pathway of admission.

**TABLE 3:** Sample characteristics—demographics and self-reported clinical and service use data (*n* = 14).

Characteristic		Participants [*n*, (%)]
Sex	Female	8 (57)
	Male	6 (43)
	*Total*	*14 (100)*
Age group	20s	2 (14)
	30s	4 (29)
	40s	2 (14)
	50s	4 (29)
	60s	2 (14)
	*Total*	*14 (100)*
Living arrangements	By themselves	11 (79)
	With others	3 (21)
	*Total*	*14 (100)*
Mental health diagnosis (main)	Depression	5 (36)
	Bipolar	4 (29)
	PTSD	2 (14)
	Anxiety	1 (7)
	BPD	1 (7)
	None, undetermined	1 (7)
	*Total*	*14 (100)*
Admission(s)	1	7 (50)
	2	6 (43)
	3	1 (7)
	*Total*	*14 (100)*
First admission pathway	Step up	9 (64)
	Step down	5 (36)
	*Total*	*14 (100)*
Completed program (4 weeks)	Yes	8 (57)
	No	6 (43)
	*Total*	*14 (100)*
Admission extended	Yes	3 (21)
	No	11 (79)
	*Total*	*14 (100)*

Qualitative results as they relate to the topic of this paper are presented below, with supporting quotations (contextualized with an indication of interview number, gender, age group and referral pathway) shown in Table 4.

**TABLE 4:** Supportive quotations for each topic and theme.

Topic	Theme	Quotation
Daily activities	Flexibility vs. lack of structure	“I didn’t feel pressure to do things. Like there was obviously a bit of—not pressure, but encouragement. They want us to be there. They want us to—it’s a two-way street, you know, you participate as well. So it was gentle encouragement, but it never felt like I was forced to do things I didn’t want to” (I13; male; 40s; step-down).
		“I could have my car and I could go to my mum’s, because she was moving, so you have freedom as well; you’re not trapped. It’s not under lock and key” (I01; female; 50s; step-up).
		“There’s nothing structured. It was sort of like up to you to figure out what you’re going to do […] Most people I suppose, don’t like classwork, but it’s structured, and it’s a way that, it doesn’t force you to do the work, but it assists you to do the work. You know, like you can go and sit in your room, or you can go sit on the verandah, or you can go and sit in the TV room, or you go sit in the art room, on your own, with the information, with the pamphlets, but you have to be independent, and you have to have the strength, you have to have the motivation, you have to have the inclination” (I12; male; 40s; step-up).
		“Just coming from rehab, everything was structured and mandatory, which works in the way of being able to learn and help yourself. Whereas I went in there and I just basically did what I did at home” (I09; female; 20s; step-up).
	Group sessions	“I found myself again. I had a voice. And no-one would say ‘hey’. No-one would criticise you. No-one criticises anyone in here. That’s very important” (I02; male; 50s; step-up).
		“I don’t know if they could have someone with you, like an extra person with you, and sit with you in the class the whole time and go through it with you. Because I did have people sitting next to me to spell out things for me sometimes. That’s where I felt really embarrassed with other people in the room. It made me feel embarrassed. Sometimes I would sit there, and I would look at it, but I wouldn’t do some of it because I couldn’t understand it. Then I felt too embarrassed to actually ask” (I04; female; 50s; step-up).
	Safety and the physical environment	“They had to have someone to let people in the door. You had your own room, and you locked your room at night, so you knew you were safe. You knew there was someone there at night-time that you could call if you needed to, so you felt safe that there was someone there, on the premises” (P04; female; 50s; step-up).
		“I felt really safe. Like, if you don’t want to see anyone, they won’t let them in, and that was very important because I know I’m a man and that, but you do get frightened sometimes when you’re vulnerable” (I02; male; 50s; step-up).
Interactions with staff and peers	Support and care	“It was like the people that were here, the two times, it was like we’d known each other for years. It was like family. Whereas your own family didn’t really give a toss” (I01; female; 50s; step-up).
		“I did get on really well with the […] staff. They were very understanding, I felt, and very kind and caring. You felt like you could go and open up to them at any time” (I04; female; 50s; step-up).
		“The staff that were there, they were sort of, I don’t know, supervising, more of a role of supervisors, just maintaining—they weren’t really there to offer any guidance, other than just to listen” (I12; male; 40s; step-up).
		“It was helpful to go and talk, and some of the subjects that came up, and you talked about—you sort of opened up to the people that were in the room there as well, and you talked. And you found that you weren’t the only person going through it. That’s what I found helpful. At different stages it was hard at times to talk about things that had happened. But you felt better after actually saying it and getting it out” (I04; female; 50s; step-up).
	Group dynamics	“Some of them are the nicest people I’ve ever met. Some really fabulous women, really caring. Like I said, they spend hours with you. Like G Ward, they just hand you pills and ignore you all day. And the staff in there are really, really fantastic but it only takes one member that everyone’s terrified of. And there’s the other dynamic where—so there’s six of you, now she likes two of you. Two of you she treats nicely, and you’ve got to watch this. And you’ve got to watch you being one of the other four”(I06; female; 60s; step-up).
Discharge process	Readiness	“I said ‘look, I don’t feel ready. I know it in myself’. They said ‘look, you can’t stay any longer, you’ve got to go’. But they said ‘you can come back after so many days’. But I just battled on at home. I was getting worse and worse. So that’s why I ended up in the mental health ward” (I04; female; 50s; step-up).
		“I had the feeling that I knew I’d outgrown [the service] after 2 weeks—but that’s just me, that’s not everyone. I’d got what I needed from the program and I was starting to feel like running away. I was starting to feel like just packing my car and taking off in the middle of the night. So, things were getting to me there. The environment was too trapped, and that meant I was getting well” (P07; female; 50s; step-down).
		“Just given I guess everything that’s happened since then, looking back now at the time between when I was discharged and then when I was re-admitted to hospital back in August last year, I wasn’t right. And I thought I was, but I wasn’t really. So, I probably rushed it a bit too much” (I13; male; 40s; step-down).
	Community support	“After my second trip to (the service), they did organise some support for when I went home, which they did not do and they did not follow through with anything like that the first time around.”(I06; female; 60s; step-up).
Follow-up post-discharge	Follow-up phone calls	“It was a helpful reminder that there was this option, I guess. If things did get bad again, then I could always come back here” (I13; male; 40s; step-down).
		“They rang a couple of times to see if I was all right, and then I’d ring here. There was always that support […] I feel I could always come back here and chat with them if I really needed just someone to talk to” (I01; female; 50s; step-up).
		“They said they’d do a follow up phone call on three days, or something, after I left. Didn’t happen. Then they said they do another follow up phone call a week—that happened, but it was just token. It wasn’t really—not like you are genuinely asking questions. It was, So, everything’s fine there? So, yes, you’re going good, are you? Yes, yes. Oh, that’s good” (I07; female; 50s; step-down).
		“You could have said, ‘I’m going great’ or ‘I’m going terribly’. Nothing would have followed from it” (I06; female; 60s; step-up).
	Views on additional services	“One thing I would like is for [the service] to do a one-month or three-month on after you’ve left barbecue and invite all the people that were at [the service] at your time to a barbecue and just—[…] so you can see them all again and catch up with everyone” (I10; male; 30s; step-down).
		“It’s all very well to advertise to people a program that presents that when you leave, we will follow up with you. It is not what happens. And one of the staff members even said to me ‘Yep, we’ve got you in here and we build you up and pad you up and tell you you’re fantastic and you’ve got all these people around. And then we send you out the door and you’re on your own” (I06; female; 60s; step-up).
		“Even if it was a follow-up as, like what we learnt and the programs that we put in place, as like the follow-up of: ‘OK, how are you doing? What are you doing now? How’s it working out for you? Is there anything you want to work on? Is there anything that needs fine-tuning? Is there anything that we can do?’ So, I suppose for me it’s reconnecting back and linking in, instead of just being kicked out the door and that’s it.”
		“It’d be very easy for people to get so attached to the support that they fall apart as soon as they leave the facility, but for me I decided that no, I didn’t want to sort of attach myself to those people any more than necessary, so that I could manage when they’re not around” (I14; male; 60s; step-up).
Life after discharge	Recovery goals	“I managed to go and get my own place, move to Perth, get a job, my old job back. I wouldn’t have been able to do that if I hadn’t come here” (P01; female; 50s; step-up).
		“I decided that I wanted to not worry about getting a job pronto. Do some volunteer stuff maybe and work on rebuilding the relationship with my kids, get to know my grandchildren” (I06; female; 60s; step-up).
	Support networks	“I have Chorus, and DSN, which is the Depression Support Network. I have a coffee group. Then one Thursday you go on picnic, the following Thursday you go out on a luncheon and you go for a trip. It just depends on where they’re taking you at the time” (I04; female; 50s; step-up).
		“I’ve got a three-grand grant with it to fill up the house with furniture. Basically my routine is apply to any job that I can because I’m allowed to work a couple of days a week. I’m on the disability pension, so I’m allowed to work a couple days a week. I haven’t been able to find any work yet, but I’m always on the lookout. Because otherwise the weeks are so long, and you can fall into bad habits when you live based off—you know, I still had a cigarette, smoke or drink and—I don’t want to sit down and just drink or smoke all day. I want to win, you know? I want to win. So it’s been good. I go to the gym every morning” (I10; male; 30s; step-down).

#### 3.4.1. Daily life and activities

Participants reported that there were no mandatory activities—with the exception of meal preparation, to which they were expected to contribute several times a week. Attendance at the morning group sessions was encouraged but not mandatory, and participation in the afternoon activities was optional, with some describing the afternoons as “free time.” The flexibility of the program was experienced in different ways; whilst some appreciated the freedom and the ability to make their own decisions, others expressed a preference for more structure.

Among those who valued the program’s flexibility, some highlighted that they did not feel “forced” to take part in any activity. Having the ability to have their car, drive places and visit family was also valued positively, as was the ability to pursue part-time work, which several participants reported doing during their stay at the service. Overall, step-down participants tended to value the flexibility of the program more positively. In contrast, other participants expressed a preference for more structure and more group work, and there was a perception that the program relied too much on self-motivation and personal initiative.

Although not compulsory, all participants reported attending the morning group sessions (which they referred to as “classwork”) regularly. These were highly valued and participants reported that they helped in building self-esteem and confidence. Although most participants valued the opportunity to share their experiences in a group setting and they acknowledged that staff were available to support and guide them through the activities, some reported struggling because they were not “academic,” and some found some of the self-reflection activities confronting.

The physical environment and layout of the facility (with a garden, a communal kitchen, an art therapy room and individual ensuite rooms) were valued positively by all interview participants. Safety was a critical component of the physical environment, especially for those who reported having been the victims of domestic violence, and participants appreciated features such as the ability to lock their own bedroom door, having someone on site at night, and the front door being locked 24 h a day. All agreed that they had felt safe during their stay, and some referred to the service as their “safety zone.”

#### 3.4.2. Interactions with staff and peers

Participants’ lived experiences of the service were strongly influenced by their interactions with staff and fellow consumers. Those reporting an overall positive experience tended to describe their relationships with staff and peers with affection, in some cases describing them as “family.” Consistent with qualitative data from the Exit Questionnaire, relationships with staff were among the most highly valued aspects of the program. Participants valued personal attributes such as being understanding, welcoming, open, caring and non-judgmental. The majority of participants valued that staff were available and that they could talk to them at any time, however, there was a perception that these conversations had to be self-initiated, which was not always experienced in a positive way.

Most participants acknowledged the therapeutic effect of sharing their lived experiences with others, especially during the group sessions. Although many found comfort in the knowledge that their fellow residents shared similar lived experiences, there was evidence that these sessions could also bring up confronting issues, and some participants reported feeling confronted when listening to accounts of domestic violence or witnessing signs of self-harm (scars).

There was some evidence that the size and gender composition of the group could affect group dynamics. One female participant reported feeling uncomfortable in a group of much younger women, whilst a male participant noted that during his stay all other residents were female and he felt “he didn’t have any mates to talk to.” There were also some reports of cliques forming and perceived staff favoritism toward specific residents. Further, there was some evidence that negative interactions with staff could lead to consumers leaving the service without completing the program. Although rare, recollections of experiences of negative interactions with staff members triggered emotional reactions among a few participants, who tended to use emotive language to describe them.

#### 3.4.3. Discharge process

All participants reported having an understanding that the program had a duration of 4 weeks, unless an extension was granted (in our sample, three participants reported having been granted an extension). The majority of participants reported feeling anxious in the days leading up to their discharge, and reports of attitudes toward readiness for discharge varied depending on whether or not they had been readmitted—half the sample (*n* = 7) reported multiple admissions (six reported two admissions and one reported three).

There was a perception among most of those who had been readmitted that they were not ready to be discharged at the end of their first admission, and there was evidence suggesting that, in some instances, lack of community support following discharge had contributed to their re-admission. One participant explained that she had no support at home except for her general practitioner and her counselor, and she had felt “dumped by everyone.” She explained that she had ended up being admitted to the mental health unit at the local hospital before being readmitted to the service. Another participant reported being referred to a local drug and alcohol service following his first discharge. He explained that they had been good at addressing his substance use problems, but they could not do anything for his mental health issues, adding that this had been the reason why he had been readmitted to the service. One female participant cited going back to an abusive domestic situation as the reason for the aggravation of her anxiety, which led to her second admission, whilst another participant spoke of “falling off the rails” after his first discharge, reflecting on how he had got more from the program the second time around.

#### 3.4.4. Follow-up after discharge

The majority of participants (*n* = 10) reported receiving two or three telephone calls following discharge, whilst the remainder (*n* = 4) reported not receiving any. Among those who received follow-up calls, some found them helpful, as they were a reminder that the service was there if they needed to reach out in the future. In contrast, others experienced the calls as tokenistic.

When asked to identify additional services the organization could provide, participants suggested organizing social events for former consumers and their families and careers to catch up and support one another in their recovery journey. There were also calls for the establishment of outreach services and follow-up sessions to make sure former consumers were making progress toward achieving their recovery goals; however, participants acknowledged that these additional services were out of scope for the organization under the current funding model. Several participants commented on the lack of services available outside of the main town, and it was thought that any future outreach programs would especially benefit those who did not have any other community services available and who might otherwise feel left to their own devices following discharge.

#### 3.4.5. Life after discharge

Participants’ reports of life after discharge showed that the majority were striving toward achieving their recovery goals, as they spoke of working or looking for work, reconnecting with family (including enjoying their grandchildren), reconnecting with friends and church communities, and finding secure accommodation.

Most participants reported being currently linked in with support services, including local community mental health services, alcohol and drug services, social and employment services, and support groups. Those who reported more complex psychosocial issues, especially a recent history of domestic violence, tended to be ones describing more uncertain current circumstances, and one participant, for example, explained that she was about to be evicted from her rental accommodation as she was in arrears with her rent. Overall, participants acknowledged that they had good days and bad days, but their narratives suggested that most had made progress toward achieving their recovery goals and they had hope for the future.

## 4. Discussion

Our results demonstrate a high level of consumer satisfaction with the service and show that, overall, admission to the service resulted in positive outcomes for consumers. Our data also highlight some challenges which need to be interpreted in the context of organizational adjustments as the service completed its implementation stage; many of these challenges are exacerbated by the rural context in which the service operates and have commissioning and funding implications.

Admission to the service was associated with improvements in consumers’ self-reported psychological wellbeing, self-efficacy, and work and social adjustment levels; this finding is consistent with existing evidence (7), including evaluation data from a larger Western Australian suburban SUSD service (14) and evaluation data from similar services in regional New South Wales (7), North Queensland (16), and Victoria (13) A review of community mental health care programs in Australia identified benefits of programs that incorporated case management and therapeutic elements, and a multidisciplinary team, in delivering positive clinical and psychosocial outcomes (31). SUSDs occupy a space between community and in-patient care; however, our findings are consistent with this broader review of Australian services.

The overall high level of satisfaction with staff is pleasing, and our results on staff characteristics that are valued by consumers (such as being caring, supportive and approachable) are consistent with evaluation data from urban SUSD services (18). Our data also show that the reduced group and the small size of the facility may pose additional challenges to group dynamics among consumers, and between consumers and staff, which may require additional formal clinical staff supervision and enhanced staff training.

Our results on the flexibility of the program and the program activities are mixed. Whilst survey data shows overall satisfaction with the program, our interview data suggest that the flexibility of the program might not suit all consumers and raises the issue of how to scaffold consumers’ recovery whilst remaining focused on individually driven recovery goals and strategies. Further, the data lend support to existing evidence suggesting that the needs of step-up vs. step-down consumers may vary (32), and should guide program delivery and content.

Our data show low representation from culturally and linguistically diverse (CALD) populations among service users. One explanation may be that they are under-represented in the referring services, however, this finding warrants further investigation, given that the region has a significant CALD community. Aboriginal and Torres Strait Islander people were also under-represented among consumers, despite Indigenous Australians experiencing higher rates of mental health issues than non-Indigenous Australians (33). Although Indigenous representation was higher than that noted in an urban WA site (14), the question remains regarding the service’s cultural safety and accessibility. Accessibility for at-risk groups in rural settings requires the specific attention of service planners, purchasers and providers if population outcomes are to be improved in these communities. Social and emotional wellbeing models of mental health service delivery for Indigenous Australians that embrace more traditional holistic models of care embedded within culturally safe and responsive practice and environments show promise in achieving more equitable mental health outcomes for improving recovery outcomes (34). Such a model is currently being implemented by the service and should result in greater engagement with Indigenous services and Indigenous consumers. Our interview data showed that consumers valued the physical environment and its personal safety aspects highly. Cultural and spiritual needs were explored during the interview and this exploration did not yield any significant data, however, a further examination of the cultural safety of the service is warranted, especially given that, having formerly housed the hospital’s palliative care, the grounds are not considered culturally safe from an Aboriginal perspective.

From a policy viewpoint, the limited correlation between consumer satisfaction with the service and change outcomes warrants further investigation, given the increasing recognition of the importance of person-centered service and a service co-design approach.

Administrative and interview data show a high number of re-admissions to the service, which raises questions about the adequacy of the program duration. Our data suggest that the 28-day admission (as stipulated by the current funding model) might be insufficient for a third of consumers. Inevitably, the complexities of commissioning, funding and consumer needs are too simply expressed in the expectation that a 28-day admission should suffice for a majority of consumers to complete a component of their recovery journey to improve their functioning and achieve their recovery goals. However, it is worth noting that, while state-level program descriptions frequently indicate a length of stay of up to 28 days, a study of 19 metropolitan and rural SUSD services in Victoria identified a mean length of stay of 18, with wide individual variation (4). The causes driving the need for an extended admission remain unclear and warrant further research. This issue may be more salient in rural settings, given the comparative paucity of community services and resources available to consumers following discharge. In the absence of options for alternative interventions in rural settings, a case can be made that the 28-day admission duration standard may not be fit for purpose for a significant proportion of consumers. Further research is needed to understand the reasons for this finding.

Our findings relating to the post-discharge period need to be interpreted in the context of the gaps in the ecosystems of support in the region, with scarce, if any, services available outside of the main town. These findings suggests that the “social interface” domain, that is, the degree to which services facilitate community linkages for consumers (4) is more salient in rural settings, given the constrained local resources. Lack of integration with community services may lead to SUSD admissions seen as “an episode of care” isolated from the rest of the consumers’ care and recovery plans. Whilst service-level adjustments may prove effective (for example, developing the discharge recovery plans throughout the admission, emphasizing on-going opportunities to “check in” on plan activation and utility), our results suggest that there is a need for follow-up support to be considered as an integral part of service delivery in rural settings. This is a systemic issue that ought to be addressed by commissioning bodies as post-discharge support is currently out of scope.

The study took place across the first 2 years of service delivery implementation and, thus, needs to be understood within this context. It could be argued that staff enthusiasm and the embedded peer support model might have had a positive effect on evaluation results; however, we speculate that this would have been countered by teething issues including staff turnover. Staffing and clinical supervision issues need to be understood in the context of the shortage of the rural health care workforce and training issues affecting Australia, exacerbated by the effects of COVID-19 travel restrictions (35). Nevertheless, our data suggest that service commissioning and funding for sub-acute services in rural areas need to consider scalability issues, recognizing that smaller scale rural services are likely to require more resources per capita compared with larger urban sites.

We acknowledge some study limitations. Data available only showed the most recent record of each consumer, regardless of the number of admissions; this limitation may originate from the service data management and/or extraction system. As a result, potential rich information on repeat consumers (e.g., dynamic characteristics such as co-morbidities and age, as well as improvement or progress over time) could not be examined. With regards to the qualitative component, our findings are based on a small sample size which included a relatively large proportion of male participants. Also, we did not include the views of potential consumers who had not been admitted to the program and thus, could not explore their unmet needs. Further, the possibility of recall bias cannot be excluded, and recall issues may also have been impacted by any emotional distress experienced during the interview.

Our results suggest that outcomes for consumers may be enhanced by implementing flexible approaches that address some of the resource limitations of the rural context. Perhaps one of the lessons is that the current funding model for SUSD services in Australian rural settings may not be fit for purpose. In developing sub-acute mental health services for rural communities, more needs to be understood about the role these services play in small regional locales and how they fit in the local rural mental health care ecosystem, how they can be better integrated with existing support services, and how they can be better funded so they can deliver responsive, effective services that are consumer-focused and aligned with recovery-oriented principles.

## Data availability statement

The raw data supporting the conclusions of this article will be made available by the authors, without undue reservation.

## Ethics statement

Ethics clearance was granted by the University of Western Australia Human Research Ethics Committee (Ref RA/4/20/6418) and the Service Research and Evaluation Committee. The participants provided their written informed consent to participate in this study.

## Author Contributions

HN carried out the analysis of the quantitative data. BC-B conducted the analysis of the qualitative data in collaboration with RB and MC. All authors contributed to the development of the manuscript, discussion and interpretation of results, and discussion and development of the final manuscript draft.
